# Altered structural connectome of children with auditory processing disorder: a diffusion MRI study

**DOI:** 10.1093/cercor/bhad075

**Published:** 2023-03-16

**Authors:** Ashkan Alvand, Abin Kuruvilla-Mathew, Reece P Roberts, Mangor Pedersen, Ian J Kirk, Suzanne C Purdy

**Affiliations:** School of Psychology, Faculty of Science, The University of Auckland, Auckland central, Auckland 1010, New Zealand; Eisdell Moore Centre, The University of Auckland, Auckland central, Auckland 1023, New Zealand; School of Psychology, Faculty of Science, The University of Auckland, Auckland central, Auckland 1010, New Zealand; Eisdell Moore Centre, The University of Auckland, Auckland central, Auckland 1023, New Zealand; School of Psychology, Faculty of Science, The University of Auckland, Auckland central, Auckland 1010, New Zealand; Centre for Brain Research, The University of Auckland, Auckland central, Auckland 1023, New Zealand; School of Psychology and Neuroscience, Auckland University of Technology, Auckland central, Auckland 0627, New Zealand; School of Psychology, Faculty of Science, The University of Auckland, Auckland central, Auckland 1010, New Zealand; Eisdell Moore Centre, The University of Auckland, Auckland central, Auckland 1023, New Zealand; Centre for Brain Research, The University of Auckland, Auckland central, Auckland 1023, New Zealand; School of Psychology, Faculty of Science, The University of Auckland, Auckland central, Auckland 1010, New Zealand; Eisdell Moore Centre, The University of Auckland, Auckland central, Auckland 1023, New Zealand; Centre for Brain Research, The University of Auckland, Auckland central, Auckland 1023, New Zealand

**Keywords:** auditory processing disorder, structural connectivity, diffusion MRI, graph theory, rich-club

## Abstract

Auditory processing disorder (APD) is a listening impairment that some school-aged children may experience despite having normal peripheral hearing. Recent resting-state functional magnetic resonance imaging (MRI) has revealed an alteration in regional functional brain topology in children with APD. However, little is known about the structural organization in APD. We used diffusion MRI data to investigate the structural connectome of 58 children from 8 to 14 years old diagnosed with APD (*n* = 29) and children without hearing complaints (healthy controls, HC; *n* = 29). We investigated the rich-club organization and structural connection differences between groups. The APD group showed similar rich-club organization and edge-wise connection compared with the HC group. However, at the regional level, we observed increased average path length (APL) and betweenness centrality in the right inferior parietal lobule and inferior precentral gyrus, respectively, in the APD group. Only HCs demonstrated a positive association between APL and the listening-in-spatialized-noise-sentences task in the left orbital gyrus. In line with previous findings, the current results provide evidence for altered structural networks at the regional level in the APD group, suggesting the involvement of multimodal deficits and a role for structure-function alteration in the listening difficulties of children with APD.

## Introduction

Auditory processing disorder (APD) is a term used to describe children who experience atypical difficulty in understanding speech or other complex auditory stimuli, particularly in the presence of background noise, or in quiet; APD is diagnosed using a battery of clinical tests (American Speech-Language-Hearing Association (ASHA) 2005; Dawes et al. 2008; Sharma et al. 2014; Keith et al. 2019; Dillon and Cameron 2021). It is estimated that globally 5.1% and locally (i.e. in New Zealand) 6.2% of school-aged children have APD-related difficulties understanding speech in the classroom despite showing normal hearing sensitivity based on the pure tone audiogram (Hind et al. 2011; Purdy et al. 2018; Keith et al. 2019). This listening difficulty is believed to arise from the complex processing of auditory information in the central auditory nervous system (CANS) in conjunction with other sensory and higher-order brain network processing involved in language, hearing, auditory, attention, and memory (Ponton et al. 1996; American Academy of Audiology (AAA) 2010; Moore 2012; Wilson 2018; Keith et al. 2019; Dillon and Cameron 2021). APD is heterogeneous and can co-occur with other neurodevelopmental disorders such as attention-deficit/hyperactivity disorder (ADHD), autism spectrum disorder (ASD), dyslexia/reading disorder, and specific language impairment (Dawes et al. 2009; Sharma et al. 2009; Halliday et al. 2017; Gokula et al. 2019; Mealings and Cameron 2019). It is anticipated that 40–56% of children diagnosed with APD also have other comorbid conditions (Ahmmed et al. 2014; Gokula et al. 2019). The overlap of APD symptoms with other sensory or cognitive neurodevelopmental disorders has raised questions about whether APD solely arises from atypical auditory sensory processing (bottom-up approach: related to the ear or CANS) or whether cognitive differences also contribute (top-down approach to cognitive function from multimodal processing) (Dawes and Bishop 2009, 2010; Dillon et al. 2012; Moore 2012, 2018; Cacace and McFarland 2013; Moore and Hunter 2013; McFarland and Cacace 2014; Iliadou et al. 2018; Dillon and Cameron 2021). It has been suggested that utilizing neuroimaging approaches may help researchers and clinicians to differentiate the neural mechanisms underlying APD (AAA 2010; Bartel-Friedrich et al. 2010; Schmithorst et al. 2011; Moore and Hunter 2013).

In the past few decades, diffusion tensor imaging (DTI) has been widely used to study white matter (WM) microstructural changes in neurodevelopmental disorders (Ameis et al. 2011; Billeci et al. 2012; Ercan et al. 2016; Beare et al. 2017; Sihvonen et al. 2021). Due to its sensitivity to microstructural tissue properties, DTI can be used as a clinical tool to study WM anatomy and the brain’s structural connectome by providing fiber orientation and quantitative diffusion measures such as fractional anisotropy (FA) and mean diffusivity (MD), as well as the axial and radial diffusivity (AD, RD) (Beaulieu 2002; Soares et al. 2013). These measures have been previously used to study auditory pathways in children with sensory processing disorder (Owen et al. 2013; Chang et al. 2014) and congenital sensorineural hearing loss (SNHL) (Huang et al. 2015; Park et al. 2018). In the APD literature, to our knowledge, two studies have investigated WM microstructure in APD by assessing the association between DTI scalar measures and APD diagnostic test variables (Schmithorst et al. 2013; Farah et al. 2014). Early research (Schmithorst et al. 2013) on 24 children diagnosed with (*n* = 10) or without (*n* = 14) APD investigated whether right or left ear advantage scores on dichotic listening tests (REA/LEA), indicators of hemispheric dominance for auditory processing, language, and learning disorders, can be predicted using DTI and functional magnetic resonance imaging (MRI) techniques. Their results showed there was greater AD in the sublenticular part of the left internal capsule in the APD group compared with healthy controls (HC). A follow-up DTI study by the same research team was conducted on 12 children with APD and HC (*n* = 12) to identify biomarkers of listening difficulties based on WM microstructures (Farah et al. 2014). Their results showed that for the APD group who had LEA, there was reduced FA in the bilateral prefrontal cortex and left anterior cingulate and increased MD in the posterior limb of the internal capsule. Results based on both studies suggested that listening difficulties in children with APD are associated with altered WM microstructure, with sensory and supramodal differences underlying the group differences in auditory processing performance. Although these studies have utilized DTI metrics on children with APD to investigate the relationship between measures of listening difficulties and WM microstructures, no study has yet reported the brain structural connectome in children with APD.

In recent years, network neuroscience has become a promising tool for studying the complex network topology of the brain (Bullmore and Sporns 2009; Sporns 2011; Bassett and Sporns 2017). This method utilizes graph theory by modeling the brain as a network composed of nodes and edges to investigate brain structural and functional connectome (Bullmore and Sporns 2009). Within this conceptual framework, brain topological properties can be studied to characterize atypical brain network topology in brain diseases and disorders (Meunier et al. 2009; Rubinov and Bullmore 2013; Crossley et al. 2014; DeSalvo et al. 2014; Yuan et al. 2015; Li et al. 2016; Fang et al. 2020; Roger et al. 2020; Lu et al. 2021; Alvand et al. 2022). Studies have revealed that the brain hub structure shows densely interconnected and rich organization within hub regions, called the rich-club phenomenon (van den Heuvel and Sporns 2011; Pedersen and Omidvarnia 2016). The rich-club is a hierarchical organization where hub regions (i.e. core regions) tend to link more densely among themselves than peripheral regions, and provide interregional brain communication and integration and enable global neural signaling (Zhou and Mondragon 2004; Colizza et al. 2006; Opsahl et al. 2008; van den Heuvel and Sporns 2011). The rich-club topology can provide important information about integrated communication in brain networks (Colizza et al. 2006; van den Heuvel and Sporns 2011). Consequently, studying the topological architecture of a rich-club organization could uncover pathological bases for brain diseases (Van Den Heuvel et al. 2013; Daianu et al. 2014, 2015; Shu et al. 2018; Xue et al. 2020; Liu et al. 2021; Lu et al. 2021) and disorders (Ray et al. 2014; Keown et al. 2017; Lou et al. 2021; Wang et al. 2021; Cui et al. 2022). A recent connectome-based study of children with SNHL compared with HC revealed alterations in the rich-club organization in children with SNHL (Cui et al. 2022). Thus, investigating the brain hub and rich-club organization of children with APD may advance our understanding of the pathobiology of this neurodevelopmental disorder.

In the present study we investigated large-scale WM network organization of children with APD using graph-theoretical analyses. We utilized diffusion MRI (dMRI) data to construct the brain structural network to explore the brain hub and rich-club architecture of children diagnosed with APD and HC. Additionally, we assessed the structural connectivity differences between these two groups. In a recent resting-state functional MRI study (rsfMRI) on children with and without a diagnosis of APD (Alvand et al. 2022), we investigated brain functional hub topology. Our study suggests that functional brain networks in APD were similarly integrated and segregated compared to HC but were significantly different within the default mode network (DMN) in bilateral superior temporal gyrus (STG). Similar to our previous research with functional brain imaging, we hypothesized that the structural connectome does not differ between HCs and children with APD on a whole-brain level. Nonetheless, the brain’s WM may be affected within specific regions involved in auditory and related processing functions.

## Materials and methods

### Participants

A total of 66 children aged 8–14 years were recruited for this research as part of previous research (Alvand et al. 2022); eight participants were excluded from this analysis due to incomplete scans (*n* = 4) or head motion (*n* = 4). Of the remaining participants, 29 were diagnosed with APD (14 boys, Age = 10.89 ± 1.53) and 29 were HC (14 boys, Age = 11.93 ± 1.41). Children diagnosed with APD were recruited from the SoundSkills clinic (https://soundskills.co.nz) in Auckland, New Zealand, based on New Zealand’s standard guidelines for APD test batteries (Keith et al. 2019). Children in the HC group were recruited via flyers and online advertisements based on the absence of hearing loss or hearing difficulties, neuropsychiatric disorders, or medication affecting the central nervous system. In the APD group, 11 children were also diagnosed with comorbid disorders such as ADD/ADHD (*n* = 2), dyslexia (*n* = 8), and developmental language disorder (DLD, *n* = 1). In the HC group, four children were diagnosed with comorbid disorders such as ADHD (*n* = 2), dyslexia (*n* = 1), and ASD (*n* = 1), but they were not experiencing any hearing or learning difficulties. These comorbidities were not excluded as they coexist with APD (Sharma et al. 2009; Dawes and Bishop 2010; O’Connor 2012). This study was approved by the University of Auckland Human Participants Ethics committee (Date: 2019 October 18, Ref. 023546). Before completing any testing or brain imaging, children and their parents consented to participate in the study. They received financial vouchers to compensate for their participation.

#### Procedure

Our previous study described all pediatric recruitment procedures (Alvand et al. 2022). In summary, children and their parents were invited to attend two individual sessions daily to complete hearing assessments and MRI scans. For the first session, children were tested for hearing acuity, middle ear disease, and atypical ipsilateral middle ear muscle reflexes using otoscopy, pure tone air conduction audiometry (PTA), and tympanometry (Roup et al. 1998). All children had a PTA threshold of ˂20 dB HL at octave-interval frequencies from 0.25 to 8 kHz in both ears. Tympanogram results showed static admittance in the range of 0.2–1.6 mmho, with peak pressure between −100 and +20 daPa, indicating normal middle ear function. Children were also administered the listening-in-spatialized-noise-sentences (LiSN-S) (Cameron and Dillon 2007, 2008), which assesses their ability to hear and remember the target sentences in the presence of competing noise and distraction. More details regarding the derivation of LiSN-S sores were provided by (Besser et al. 2015; Alvand et al. 2022). Participants were asked to attend ⁓24-min MRI scan for the second session, including a T1-weighted image (T1w), rsfMRI (see Alvand et al. 2022), and dMRI. During T1w and dMRI sequences, participants were asked to stay still and not to move their heads or laugh while watching the movie. Earplugs and headphones were also provided to decrease the loudness of the scanner noise.

### Data acquisition

All MRI scans are acquired on the same 3T Siemens scanner SKYRA and 20-channel head coil at the Center for Advanced MRI (CAMRI), the University of Auckland. Initially, a high-resolution structural T1w (4 min; 36 s) was acquired for co-registration and parcellation using a magnetization-prepared rapid acquisition gradient echo sequences (isotropic resolution = 1 mm, field of view; FOV = 256 mm, slices = 208 sagittal slices in a single slab, repetition time; TR = 2,000 ms, echo time; TE = 2.85 ms, flip angle = 8°, slice thickness = 1 mm). Diffusion MRI was acquired using single-shot echo-planar imaging (EPI) sequences (TR = 5,000 ms, TE = 63.80 ms, FOV: 240 mm, slice thickness = 2.5 mm, voxel size = 2.5 mm^3^, Slices = 60, flip angle = 90°, GRAPPA factor = 2, phase encoding direction = AP, multi-band acceleration factor = 2). dMRI images were obtained based on 64 diffusion-weighted directions with b-value = 1,000 s/mm^2^, and five interspersed scans where *b* = 0 s/mm^2^. In addition, a single *b* = 0 s/mm^2^ was obtained with a reversed-phase encoding direction for susceptibility field estimation. The total duration of the scan was ⁓11 min with 65 volumes.

### Image preprocessing and network construction

All anatomical and diffusion data preprocessing were performed using QSIprep pipeline version 0.15.3 (Cieslak et al. 2021). Preprocessing steps for T1w data included (i) correction for intensity non-uniformity (function: *N4BiasFieldCorrection)*, (ii) skull-stripping (function: *antsBrainExtraction.sh*), (iii) spatial normalization and registration to ICBM 152 (function: *antsRegistration*), (iv) brain tissue segmentation (function: *FAST*).

**Fig. 1:**
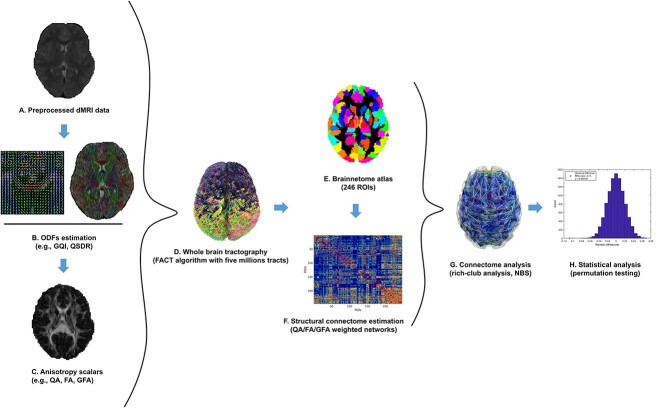
Schematic overview of the study pipeline. A) dMRI data were preprocessed using the QSIprep pipeline (Cieslak et al. 2021). B) The output from the preprocessing pipeline was then reconstructed using generalized q-sampling imaging (GQI) and the diffusion ODFs were recreated in the standard space and native space (Yeh et al. 2010). C) QA, FA, and generalized FA were estimated for individual data. D) Whole-brain tractography was carried out using a deterministic algorithm (fiber assessment by continuous tracking). E) Brain regions were parcellated into 246 ROIs using the Brainnetome atlas (Fan et al. 2016) and, F) structural connectivity was estimated using QA, FA and GFA as network edge weights. G) Next, network analysis, such as rich-club organization (van den Heuvel and Sporns 2011) and edge-wise connectivity analysis (i.e. NBS) (Zalesky et al. 2010a), were carried out. H) Finally, statistical analyses were performed on individual networks using PALM (Winkler et al. 2014).

Diffusion data were preprocessed as follows: (i) MP-PCA denoising with five-voxel window (function: *dwidenoise*), (ii) magnetic field inhomogeneity correction (function: *dwibiascorrect*), (iii) motion correction using SHORELine method (function: *3dSHORE*), (iv) susceptibility distortions correction based on two EPI references with opposing phase encoding directions (function: *3dQwarp*), (v) co-registration to T1w reference (see Fig. 1A).

Further, post-processing was performed in DSIstudio (Yeh et al. 2010), which is implemented in QSIprep (Cieslak et al. 2021). Diffusion orientation distribution functions (ODFs) were reconstructed in both native space and standard space (i.e. ICBM 152) using generalized q-sampling imaging (GQI) (Yeh et al. 2010) (Fig. 1B). Then, whole-brain fiber tracking was performed using a deterministic tractography (five million tracts) (Fig. 1D). The quantitative anisotropy (QA) scalar was calculated for defining network edges. Along with QA, FA and generalized FA (GFA) were also calculated (Fig. 1C). Brain nodes were defined according to Brainnetome parcellation with 210 cortical and 36 subcortical regions (Fan et al. 2016) (Fig. 1E). Then edge weights were computed based on the mean QA along tracks connecting any pair of regions of interest (ROIs) (Fig. 1F). The network construction resulted in an individual-specific symmetric undirected weighted connectivity matrix with dimensions of $246\times 246$.

To assess the robustness of results, edge weights were calculated based on mean FA and mean GFA. To avoid the influence of semi-arbitrary selection of parcellation, automated anatomical labeling (AAL) (Tzourio-Mazoyer et al. 2002) atlas was used to define network nodes (Zalesky et al. 2010b). This parcellation was chosen due to its similarity with the Brainnetome atlas in defining biologically meaningful boundaries for anatomical brain regions (Zalesky et al. 2010b; Wang et al. 2018a). Details on preprocessing, quality control and network construction are provided in Supplemental Methods.

### Connectome analysis

Graph theory analysis was carried out using the brain connectivity toolbox (v03/03/2019) on MATLAB R2019 (Fig. 1G).

#### Edge-wise connectivity

The network-based statistic (NBS) approach (Zalesky et al. 2010a) was conducted on an individual’s structural matrices to assess the between-group differences in the edge-wise connection. The NBS is a nonparametric statistical method that controls family wise error to identify the largest connected component in the form of alteration. Initially, a primary statistical threshold ($P<0.05$, uncorrected) was used to identify connected components and their sizes based on a set of supra-threshold links. Second, to test the significance of each identified connected sub-networks, the empirical null distribution of component size was evaluated using a nonparametric permutation test with 10,000 randomizations. Afterwards, two-sample *t*-tests were performed for each pairwise connection linking 246 brain regions to test group differences in structural connectivity in either direction (two-tailed hypothesis test, Initial *t* threshold = 3.9805). Age was controlled as a covariate.

#### Rich-club organization

The rich-club architecture in a network exists when hub nodes are highly connected, more so than expected by chance (Zhou and Mondragon 2004; van den Heuvel and Sporns 2011; Fornito et al. 2016a). To reveal the rich-club behavior of the brain networks, the weighted rich-club coefficient ${\varphi}^W(k)$ at degree level of $k$ were calculated on the group-averaged QA-weighted network (Opsahl et al. 2008). Then, the normalized rich-club coefficient [${\varphi}_{norm}^W(k$)] was calculated based on ${\varphi}^W(k)$ and average of 1,000 comparable random networks [${\varphi}_{rand}^W(k)$] (Colizza et al. 2006). The ${\varphi}_{norm}^W(k)>1$ is indicative of rich-club organization in the brain network.

Brain hub regions were identified according to the consensus-based definition of hubs in structural connectivity networks (van den Heuvel et al. 2010), whereby hubs are defined as nodes with high nodal strength (NS, top 20%), high betweenness centrality (BC, top 20%), low average path length (APL, bottom 20%), and low clustering coefficient (CC, bottom 20%). Categorization of brain regions allowed for defining the connections between brain regions into three classes: *rich* (links between hub regions), *feeder* (links between the hub and non-hub regions), and *local* (links between non-hub regions). The connectivity strength of rich, feeder, and local connections was computed for each connectome. Details regarding rich-club analysis, brain hub detection, and the strength of connectivity can be found in the Supplemental Methods.

### Statistical analysis

Differences in group characteristics (i.e. age, gender, handedness) and LiSN-S variables were tested in the Statistical Package for the Social Sciences (SPSS v28.0). A chi-square test was used to examine gender and handedness effects. Two-sample *t*-test analyses were employed to assess group differences in age and LiSN-S measures.

#### Group differences

To investigate between-group differences in the rich-club and brain hub organizations, a permutation analysis was conducted for each individual’s brain network using permutation analysis of linear models software (PALM) (Winkler et al. 2014). Two-sample *t*-tests were used assuming unequal variances for graph metrics of rich-club coefficients, normalized rich-club coefficients, and network hub measures (APL, BC, CC, NS) using 20,000 randomizations. The impact of age was controlled for as a nuisance covariate (demeaned). To control for the effect of multiple comparisons, all $P$-values obtained from graph analysis were corrected across ROIs ($k$ level for the case of rich-club metrics) and all measures [i.e. ${\varphi}^W(k)$, ${\varphi}_{norm}^W(k)$, APL, BC, CC, NS] using Bonferroni correction ($P<0.05$, PALM function: -corrmod). Between-group comparisons were also carried out to assess the differences in three classes of connectivity strength (i.e. rich, feeder, and local). A Bonferroni correction was applied to correct multiple comparisons, with the significance set at $P<0.05$.

To investigate the relationship between the network parameters that showed significant between-group differences and LiSN-S variables (z-scored), partial correlations were computed using PALM (20,000 permutations) (Winkler et al. 2014), for all participants while controlling the effect of Age. All $P$-values were corrected for the multiple comparisons problem using Bonferroni correction across ROIs and network measures (PALM function: -corrmod, $P<0.05$) (See Fig. 1H).

## Results

### Demographics

Table 1 summarizes the demographic characteristics and behavioral measures of APD and HC participants. Age differed between groups (HC$>$APD, $P<0.05$), but there were no other significant differences in the demographic and LiSN-S variables. Because of the between-group differences in age, this was included as a nuisance regressor in the statistical analyses. Apart from a statistical trend ($P=0.051$), the groups did not differ in LiSN-S performance; this test is part of a wider test battery used to diagnose APD in children. The distribution of LiSN-S variables for all participants is shown in Fig. S1.

**Table 1:** Group demographics.

	APD (29)	HC (29)	Test Statistic	${P}$ -value
Age (years)	10.90 ± 1.532	11.94 ± 1.416	2.693^a^	0.009
Gender (male/female)	14/15	14/15	0.000^b^	1.000
Handedness (right/left)	24/5	27/2	1.505^b^	0.220
LiSN-S (total/missing)	(27/2)	(28/1)		
Total advantage	0.39 ± 1.114	0.52 ± 0.991	–0.273^a^	0.652
Spatial advantage	−0.31 ± 1.525	0.24 ± 1.054	1.542^a^	0.129
Talker advantage	−0.78 ± 0.966	−0.30 ± 0.817	1.999^a^	0.051
High cue	0.13 ± 1.183	0.50 ± 0.890	1.315^a^	0.194
Low cue	−0.33 ± 1.089	0.04 ± 0.997	1.285^a^	0.204

*Note:* Data are presented as mean ± standard deviation. HC—healthy control, APD—auditory processing disorder, LiSN-S—listening-in-spatialized-noise-sentences.

^a^Two-sample *t*-tests.

^b^Pearson chi-square test.

### Whole-brain structural connectivity is unaffected in APD

The NBS analysis showed no significant between-group differences based on the QA-weighted structural connectivity. Further validation analysis based on FA-weighted and GFA-weighted networks indicated no significant between-group differences in structural connectivity between groups. These results were also consistent using AAL parcellation.

### Rich-club organization is the same across groups

Fig. 1 illustrates the rich-club organization found in the structural connectome of the APD and HC groups based on the group-averaged QA-weighted network. For both groups, the existence of the rich-club organization (${\varphi}_{norm}^W>1$) was observed over the range of degrees from $k=43-179$. Consistently, for both groups, the weighted rich-club coefficient of the empirical network was significantly higher than the weighted rich-club coefficient of random networks, based on the range of $k$, indicating robust rich-club organization in the structural network (${\varphi}^W>{\varphi}_{rand}^W$, $P<0.05$). Results from group comparisons based on ${\varphi}_{norm}^W$ and ${\varphi}^W$showed no significant differences between APD and HC individuals across $k$ levels (node degree).

**Fig. 2:**
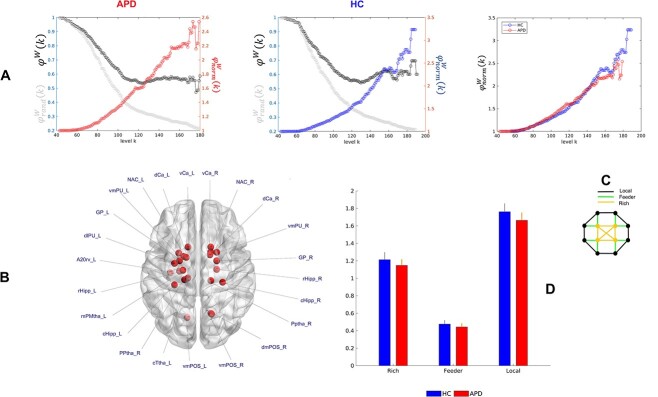
Rich-club organization in children with APD and HC. A) The empirical rich-club coefficient [black,${\varphi}^W(k)$], mean rich-club coefficient of 1,000 randomized networks [gray, ${\varphi}_{rand}^W(k)$], and normalized rich-club coefficient [${\varphi}_{norm}^W(k)$] are shown for the group-averaged networks (QA-weighted). Each symbol “o” represents a normalized/rich-club coefficient at each level $k$. APD and HC groups showed rich-club organization where ${\varphi}_{norm}^W(k)>1$. Group comparisons based on individual and group-averaged networks showed no significant differences in the normalized rich-club coefficient. B) In total 23 brain hub regions (red balls) were identified for both APD and HC groups based on the frequency of an ROI in four hub measures of NS, BC, APL, and CC. These hub/rich-club regions were found in the left fusiform gyrus, left precuneus, bilateral parietooccipital sulcus, bilateral hippocampus, bilateral basal ganglia, and bilateral thalamus. Brain hub regions were visualized in BrainNet viewer (Xia et al. 2013). See Table 2 for more information regarding brain hubs’ labels. C) The schematic view of rich connections (red) linking rich-club members, feeder connections (blue) linking hub regions to non-hub regions, and local connections (black) linking non-hub regions. D) Group comparison based on individual’s rich, feeder and local connections indicated no considerable differences between APD and HC groups. The error bars represent a 95% confidence interval of the connection strength in each group.

Results from hub detection revealed 23 hub regions (i.e. core regions or rich-club regions) for both APD and HC groups. These rich-club regions included the following ROIs according to the Brainnetome atlas: left fusiform gyrus, left precuneus, bilateral parietooccipital sulcus, bilateral hippocampus, bilateral basal ganglia, and bilateral thalamus, confirming the results of a previous report (van den Heuvel and Sporns 2011). More detailed information about hub regions can be found in Table 2. After revealing core regions (i.e. hub), peripheral areas (i.e. non-hub) were identified, and the rich, feeder, and local connection strengths were compared between groups. No significant differences between groups were found in the connectivity strength of either rich, feeder, or local connections (Fig. 2B).

**Table 2:** Brain hub regions in APD and HC groups.

ROIs	Anatomical region	Label	X	Y	Z
	**Fusiform gyrus**				
103	Left rostroventral area 20	A20rv_L	−33	−16	−32
	**Precuneus**				
152	Right dorsomedial parietooccipital sulcus	dmPOS_R	16	−64	25
	**Medio Ventral Occipital cortex**				
197	Left ventromedial parietooccipital sulcus	vmPOS_L	−13	−68	12
198	Right ventromedial parietooccipital sulcus	vmPOS_R	15	−63	12
	**Hippocampus**				
215	Left rostral hippocampus	rHipp_L	−22	−14	−19
216	Right rostral hippocampus	rHipp_R	22	−12	−20
217	Left caudal hippocampus	cHipp_L	−28	−30	−10
218	Right caudal hippocampus	cHipp_R	29	−27	−10
	**Basal ganglia**				
219	Left ventral caudate	vCa_L	−12	14	0
220	Right ventral caudate	vCa_R	15	14	−2
221	Left globus pallidus	GP_L	−22	−2	4
222	Right globus pallidus	GP_R	22	−2	3
223	Left nucleus accumbens	NAC_L	−17	3	−9
224	Right nucleus accumbens	NAC_R	15	8	−9
225	Left ventromedial putamen	vmPu_L	−23	7	−4
226	Right ventromedial putamen	vmPu_R	22	8	−1
227	Left dorsal caudate	dCa_L	−14	2	16
228	Right dorsal caudate	dCa_R	14	5	14
229	Left dorsolateral putamen	dlPu_L	−28	−5	2
	**Thalamus**				
233	Left pre-motor thalamus	mPMtha_L	−18	−13	3
239	Left posterior parietal thalamus	PPtha_L	−16	−24	6
240	Right posterior parietal thalamus	Pptha_R	15	−25	6
243	Left caudal temporal thalamus	cTtha_L	−12	−22	13

*Note:* Brain hub regions are shown according to the Brainnetome atlas (246 ROIs). Hub regions were detected based on top ROIs that scored the highest strength (degree, $>$ 1 SD above the mean), highest betweenness centrality, lowest clustering coefficient and lowest APL. MNI coordinates of hub regions are shown as X, Y, and Z. HC—healthy control, APD—auditory processing disorder, APL—average path length, ROI—regions of interest, MNI—Montreal Neuroimaging Institute.

### Nodal differences between APD and HC

**Fig. 3:**
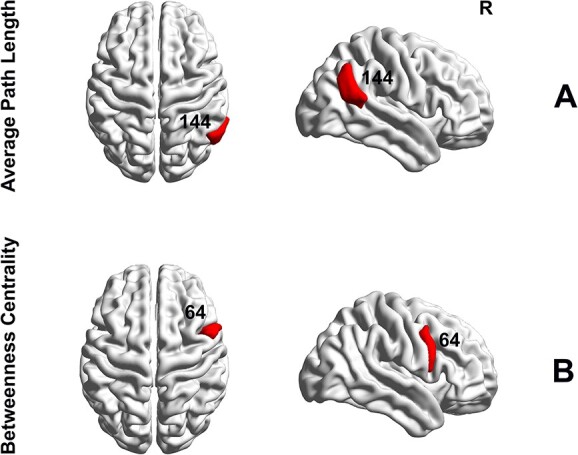
Group differences in nodal hub measures of BC and APL based on Brainnetome parcellation. A) Significant group differences in the right IPL (ROI #144) where APD showed an increase in APL ($P=0.0097$, Bonferroni corrected). B) Significant group differences were found based on BC measure in the right IPG (ROI #64, APD$>$HC, $P=0.0398$, Bonferroni corrected). Brain surfaces in sagittal and axial views were constructed in BrainNet viewer (Xia et al. 2013).

Fig. 3 illustrates nodal differences in hub measures between APD and HC groups. Group comparison showed a significant increase in APL for the APD individuals (APD$>$HC, $P=0.0097$, Bonferroni corrected) in the right rostroventral inferior parietal lobule (IPL, label = A39rv_R, ROI #144). The analysis also demonstrated significant between-group differences in BC (APD$>$HC, $P=0.0398$, Bonferroni corrected) in the right ventrolateral precentral gyrus/inferior precentral gyrus (IPG, label = A6cvl_R, ROI #64). No significant group differences were found for CC and NS measures. The data distribution of APL and BC measures across all the participants in APD and HC groups are shown in the Supplementary Figs. S2–S5.

Validation analysis based on FA-weighted and GFA-weighted networks also showed group differences in ROI #64 (based on APL measure) and ROI #144 (Based on BC measure); however, these differences did not pass the multiple comparison correction (Supplementary Table S1). Results based on AAL parcellation also did not show between-group differences in APL and BC network measures (see Table S2). Results derived from fiber reconstruction in standard space were similar to native space.

### Relationship between network metrics and LiSN-S variables

**Fig. 4:**
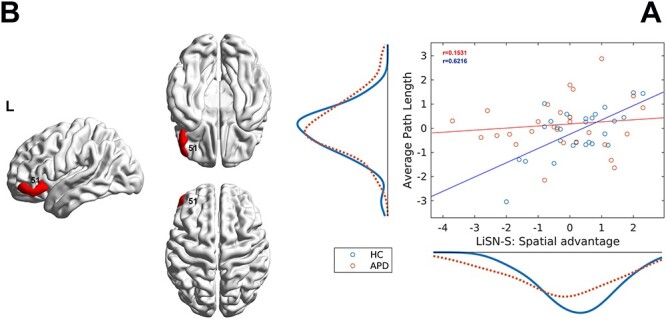
Relationship between LiSN-S variable and the network metric. A) The scatter plot shows the association between LiSN-S variable spatial advantage (z-scored) and the network metric APL (z-scored) for both APD (red) and HC (blue) groups in the ROI #51. The marginal distribution of APL (left) and spatial advantage (bottom) are also shown along axes for both groups. The solid lines represent the fitted line. The plot shows a significant positive association for the HC group in the left lateral OrG ($P<0.05$, Bonferroni corrected). B) This association is illustrated in brain surfaces in sagittal and axial views constructed by BrainNet viewer (Xia et al. 2013).

Fig. 4 demonstrates the association between the LiSN-S spatial advantage scores and the network measure of APL. The result indicated significant positive correlations between the spatial advantage and APL in the HC group in the left lateral orbital gyrus (OrG, ROI #51, Pearson $r=0.6216$, $P<0.02$, Bonferroni corrected). No significant correlation was found between LiSN-S scores and the APL network measure for the APD group.

## Discussion

In the present study, we examined brain WM connectome in children diagnosed with and without APD aged between 8 and 14 years old. This, to our knowledge, is the first study that has investigated rich-club organizations using diffusion-based connectivity and a complex network science approach. In line with our hypothesis, the current findings suggest there is a similar global WM structural connectome between APD and HC groups in terms of rich-club organization (i.e. hub topological structure), the strength of connectivity (i.e. rich, feeder, local), and edge-based connectivity. However, our regional findings (nodal measures of APL and BC) showed significant between-group differences in the right IPL and the right IPG. Additionally, the correlation analysis revealed positive associations between the APL metric in the left OrG and the LiSN-S behavioral measure of spatial advantage for HCs. These findings extend our understanding of the neuropathological mechanisms underlying APD from a perspective of the structural connectome.

### Rich-club organization is intact in APD

Rich-club organization is a key characteristic of brain networks, and its existence has not only been found during human brain development (van den Heuvel and Sporns 2011; Sa de Almeida et al. 2021), but also in numerous neurological diseases (Van Den Heuvel et al. 2013; Cao et al. 2014; Collin et al. 2014a; Ray et al. 2014; Li et al. 2020; Liu et al. 2021; Peng et al. 2021; Cui et al. 2022). In our study, the rich-club network of densely interconnected hubs was observed for both APD and HC groups in the left fusiform gyrus, left precuneus, bilateral parietooccipital sulcus, bilateral hippocampus, bilateral basal ganglia (i.e. caudate, globus pallidus, putamen, nucleolus accumbens), and bilateral thalamus. The observed rich-club regions were largely consistent with previous hearing-related research by Cui et al. (2022) for participants with SNHL and other structural brain network studies (van den Heuvel and Sporns 2011; Van Den Heuvel et al. 2013; Collin et al. 2014a). These results suggest the existence of a robust, densely interconnected rich club in the connectome for children with APD. This similarity in the rich-club organization between groups could potentially reflect intact brain WM structures in the participants with APD due to their subtle behavioral differences compared to the HC group. Additionally, the identified rich-club regions from the present study form the components of the DMN, such as the precuneus/posterior cingulate cortex (Raichle et al. 2001; Hagmann et al. 2008; van den Heuvel and Sporns 2011), which has been previously reported to have an important role in between-module connectivity (van den Heuvel and Sporns 2011) and contributes to neurocognitive functions (e.g. memory and attention) (Castellanos et al. 2008; Delano-Wood et al. 2012). Previous functional connectivity studies of children with APD reported differences in the DMN regions, such as the STG (Alvand et al. 2022; Stewart et al. 2022), and posterior cingulate cortex/precuneus (Pluta et al. 2014), indicating functional changes in the brain network.

The connectivity between the rich-club structure is hypothesized to be a foundation for high-level information transmission (van den Heuvel and Sporns 2011; Van Den Heuvel et al. 2013; Collin et al. 2014b). These dense connections enable the brain to process signals in scattered distributed modular structures and integrate the processed information across all modules through long-distance connections between hub regions (van den Heuvel and Sporns 2011). Thus, alteration in rich-club connections reflects a change in the brain’s global communications (van den Heuvel and Sporns 2011; Stam 2014). Abnormalities in the rich, feeder, and local connections have been reported previously in individuals with neurological disorders along with alteration in local topological metrics (Ray et al. 2014; Shu et al. 2018; Wang et al. 2019, 2021; Lou et al. 2021; Cui et al. 2022). In contrast, our results did not show significant between-group differences while comparing the normalized rich-club coefficient (i.e. the measure of rich-club organization) and the strength of connectivity (i.e. rich, feeder, and local connections). Still, they indicated group differences in brain networks’ local properties (i.e. APL and BC). This was inconsistent with the results from the recent hearing-related study by Cui et al. (2022), which reported an increase in local connection in SNHL and no differences in nodal topological measures. This could suggest that our results rely on differences in the topological arrangement of connections and their weights to the rich-club regions rather than the differences in the strength of such connections (Baldi et al. 2022). Hence, these findings could indicate that the information transmission between structural regions was not significantly changed between both groups. Children with APD have a similar structural connectome compared to the HC group.

### Alterations of the regional network in APD

At the local topology level, we found a statistically significant increase in the nodal measure of APL in the IPL region situated in the DMN for the APD group (ROI #144, Fig. 3A). APL is the average number of steps along the shortest path for every possible pair of nodes (Rubinov and Sporns 2010). APL measures information efficiency and higher APL suggests less efficiency in information flow (Fornito et al. 2016b). Also, an increase in APL previously reported in DTI studies of the WM connectome has been attributed to the degeneration of fiber bundles and disconnection in information transmission (Bai et al. 2012). The meta-analytical relationship between cognitive terms and the intraparietal lobule has indicated an association with theory of mind, internally oriented thoughts, and autobiographical memory (Yarkoni et al. 2011). This region is located in the DMN, which is involved in self-referential processing, higher-order cognitive functioning, and emotional self-regulation (Raichle et al. 2001; Buckner et al. 2008; Kaiser et al. 2015). Altered functional connectivity within the DMN has been reported in hearing loss (Schmidt et al. 2013) and APD studies (Pluta et al. 2014; Alvand et al. 2022). This result is our current study is in line with our previous fMRI study on children with APD, where between-group comparisons based on the participation coefficient metric indicated differences in the right IPL (ROI #298, Schaefer atlas) (Alvand et al. 2022). There have been a few other studies reported in the auditory-hearing literature identifying the intraparietal lobule in the DMN. For instance, an fMRI study of young adults with SNHL suggested a role for the IPL within DMN in linguistic thinking (Li et al. 2015). Studies of individuals with unilateral hearing loss (UHL) have also reported increased functional connectivity in this region in participants with UHL, suggesting that IPL may contribute to the remodeling of the sensory system (Xie et al. 2019). Research on UHL has also reported that IPL, an important region in the DMN, could be susceptible to chronic auditory deprivation (Yang et al. 2014).

Our results also showed a significant increase in the BC metric for the APD group in the IPG region located in the executive control network (ECN, ROI #64) (Fig. 3B). The BC metric measures the shortest paths that pass through a node (Rubinov and Sporns 2010). Nodes with a high score of BC participate in numerous shortest paths, meaning higher BC indicates greater influence and control in information transmission (Rubinov and Sporns 2010; Fornito et al. 2016c). DTI research on the WM connectome has reported that an increase in BC in a brain region indicates that the WM network associated with that region is highly affected by shorter/local pathways (Torgerson et al. 2015). Thus, our result based on BC could suggest that the IPG has a critical and central role in the structural connectome of the APD group. This area, ROI #64, is located in the ECN, which is responsible for executive functions that regulate cognitive processes such as working memory, problem solving, planning, and reasoning (Qin et al. 2015). Also, meta-analytical correlation shows that IPG is associated with working memory, demands, and tasks (Yarkoni et al. 2011). The relationship between IFG and working memory was previously reported by Kambara et al. (2017) in their study of patients with epilepsy during auditory verbal working memory tasks, which indicated the role of IPG in the initial maintenance of memory cues (Nakai et al. 2019). Adult participants with hearing loss with longer hearing aid experience showed increased right IPG activity, suggesting this region’s involvement in speech processing outside of the core speech processing network (Vogelzang et al. 2021). Consistent with this research, another study of lipreading in participants with normal hearing reported the involvement of IPG in the motor theory of speech, relating speech processing to the activation of the pre-motor region (Ruytjens et al. 2006).

On a different note, studies have shown that the DMN is an antagonist of ECN and deactivates during the activation of ECN (Barkhof et al. 2014). Neuroimaging studies of people with bipolar disorder have investigated the alteration in functional connectivity within and between DMN and ECN and suggested that this reflects dysregulation in cognitive processing (Goya-Maldonado et al. 2016; Wang et al. 2018b). The imbalance between these two networks has also as potentially associated with the self-focus condition and rumination, indicating an inability to reallocate neural resources for effective down-regulation of self-referential thoughts (Belleau et al. 2015; Kaiser et al. 2015). Thus, the results observed here for children with APD could reflect imbalance or dysregulation impacting on auditory processing.

The correlation analysis revealed a positive relationship between APL (i.e. network efficiency) and a LiSN-S variable, spatial advantage, in the left lateral OrG for the HC group, while no correlation was found for the APD group (ROI #51, Fig. 4A and B). The left lateral OrG is located in the prefrontal cortex and has been previously associated with semantics, language comprehension, sentence comprehension, and language network (Frost et al. 1999; Billingsley et al. 2001; Ferstl et al. 2005, 2008; Gitelman et al. 2005). The spatial advantage measure is part of the APD diagnostic test battery (Keith et al. 2019), and reflects the individual’s ability to use spatial cues to distinguishing the talker’s speech in the presence of a distractor (Cameron et al. 2006a, 2006b, 2011). A link between spatial processing of language and the left OrG has been previously shown in neuroimaging research (Santesso et al. 2008; Chow et al. 2014; Rocca et al. 2020).

Together our findings relative to alteration in the regional brain networks in the DMN and ECN could suggest the notion of adaptive neural response in pathological perturbation in APD, such as neural compensatory, and degeneracy mechanisms (Fornito et al. 2015). Fornito et al. (2015) explained how effectively the brain can use adaptive behavior to maintain its performance during neural insult. They specified that elevation in functional activity in neurological disorders is commonly attributed to compensatory mechanisms within the neural system, which can last for an extended period of time with a high degree of preserving behaviors (Fornito et al. 2015). This is in line with our results reported here for structural networks and our recent functional research on children with APD, which showed functional regional network differences in bilateral regions (e.g. STG and temporo-occipital cortices) (Alvand et al. 2022). Fornito et al. (2015) further explained degeneracy as a complementary to compensatory mechanisms. Degeneracy is defined as the capability of anatomically distinct regions in the brain to execute similar functions which is apparent at multiple levels (e.g. large-scale networks) (Tononi et al. 1999; Fornito et al. 2015). Multiple neural networks activate in a parallel and redundant manner during any task to support functional performance. Any possible failure in one system can be replaced by other backup systems (Noppeney et al. 2004). This is aligned with our current results regarding the increase in structural brain regions located in two segregated functional networks (i.e. DMN and ECN), suggesting a neural basis for the engagement of cognitive reserve (i.e. ability to engage alternative compensatory mechanisms to encounter behavioral changes) (Valenzuela et al. 2007; Barulli and Stern 2013; Fornito et al. 2015). Thus, our findings, combined with previous results, including our recent fMRI research, support the involvement of memory and cognitive functioning in the brain’s auditory processing network (Alvand et al. 2022) and it suggest that multimodal deficits and structure-function alterations contribute to listening difficulties.

### Limitations and future directions

This study has several limitations. First, despite reporting results based on a sample size that showed statistical effects, a larger cohort of participants should be recruited in the future to validate the findings. Second, the results from this study were based on Brainnetome structural parcellation (Fan et al. 2016). However, studies have shown that the semi-arbitrary brain parcellation selection could affect results (Zalesky et al. 2010b; Andellini et al. 2015; Termenon et al. 2016). We also calculated the results based on the AAL structural parcellation (Tzourio-Mazoyer et al. 2002) to assess the robustness of our analysis. Our results based on AAL atlas did not show between-group differences in the network measures of APL and BC. This could be due to the discrepancy between the two atlases whereby the Brainnetome parcellation contains substantially more regions than the AAL atlas, providing a detailed delineation of the brain’s cortical and subcortical regions at a finer spatial resolution. Thus, Brainnetome parcellation could more precisely pinpoint the affected brain areas in the participants with APD who were generally quite similar to the HC group. Future research could employ other parcellation schemes with a similar number of parcels to mitigate this potential issue of loss of resolution of brain regions. Third, the inherent restrictions of the dMRI sequences and deterministic tractography algorithm urge us to interpret the results carefully. The use of deterministic tractography could have influenced the results due to its limitation in resolving crossing, converging, or diverging streamlines (i.e. fiber) and producing inaccurate connectivity matrices (i.e. false positive or false negative fibers) (Maier-Hein et al. 2017). Although probabilistic tractography could potentially prevent the issue of fiber crossing, the problem of false positive fibers remains a potential issue (Zalesky et al. 2016). A possible solution to these limitations could be utilizing advanced dMRI sequences for analyzing diffusion data by fixel-based analysis (Dhollander et al. 2021). The FBA method allows the construction of fiber population within a voxel (similar to the voxel-based analysis for rsfMRI data) in the presence of complex fiber arrangements, which is considered beneficial compared to the traditional voxel averaged-approach (e.g. DTI) (Raffelt et al. 2015, 2017; Tournier et al. 2019; Dhollander et al. 2021). Using the FBA method, different diffusion indices, as opposed to the DTI/GQI method, can be obtained, such as fiber density (FD: microstructural changes in local loss of intra-axonal volume), fiber cross-section (FC: an estimate of macrostructural changes in the diameter of fiber bundle), and fiber density/fiber cross-section (FDC: the combination of FD and FC) (Raffelt et al. 2015, 2017). Future studies on the APD population can use the FBA approach to allow us to expand the understanding of the WM connectome’s microstructural and macrostructural properties. Lastly, as discussed in our previous rsfMRI study (Alvand et al. 2022), the heterogeneity of our sample could have affected the results. We recommend future research employ a longitudinal approach to investigate the developing brain of children with APD until their maturation. This is because the structural connections between brain regions are continuously growing, and exploring the rich-club organization of these changes would give us better insight regarding the brain in children with APD (Oldham et al. 2022).

## Conclusion

In summary, our study provided evidence of undisrupted whole-brain WM topological organization and abnormality in the regional structural network located in the right IPL (based on APL metric) and IPG (based on BC metric) within DMN and ECN, respectively. These brain regions are associated with cognitive functioning, such as theory of mind, autobiographical memory, and working memory. Additionally, correlation analysis with behavioral measures showed a significant positive association in the left OrG only for the HC group. Our findings could suggest the involvement of multimodal deficits and a role for structure-function alteration in listening difficulties, providing a new perspective for understanding the pathological mechanisms of APD.

## Supplementary Material

Supplementary_material_bhad075

## Data Availability

The data that support the findings of this study are available from the corresponding author upon reasonable request. No custom codes were used in any of the analyses.

## CRediT authors statement

Ashkan Alvand (Conceptualization, Methodology, Investigation, Data curation, Software, Formal analysis, Visualization, Writing—original draft, Writing—review & editing), Abin Kuruvilla-Mathew (Conceptualization, Methodology, Investigation, Funding acquisition, Supervision, Writing—review & editing), Reece R. Roberts (Methodology, Supervision, Writing—review & editing), Mangor Pedersen (Methodology, Validation, Writing—review & editing), Ian J. Kirk (Methodology, Resources, Supervision, Writing—review & editing), Suzanne C. Purdy (Conceptualization, Methodology, Project administration, Funding acquisition, Supervision, Writing—review & editing)
