# Supplementary material for: Altered structural connectome of children with auditory processing disorder: a diffusion MRI study
Source: Cereb Cortex. 2023 Mar 16;33(12):7727–40. doi: 10.1093/cercor/bhad075 (PMC10267651; doi:10.1093/cercor/bhad075)
Supplement: Supplementary_material_bhad075 [file supplementary_material_bhad075.docx]

**Supplemental Methods**

## Image preprocessing

Initially, all anatomical and diffusion images were converted to NIFTI file sets using *dcm2niix* version 11/11/2020 (Rorden et al. 2007). Then NIFTI file sets were structured according to the Brain Imaging Data structure (BIDS v1.8.2) (Gorgolewski et al. 2017). The dMRI preprocessing was performed using the QSIprep pipeline (v0.15.3, (Cieslak et al. 2021), based on Nipype (v1.7.0, (Gorgolewski et al. 2011; Esteban et al. 2022), Nilearn (v0.9.0, (Abraham et al. 2014) and Dipy (Garyfallidis et al. 2014). QSIprep is a robust integrative software platform for preprocessing and reconstructing dMRI sampling schemes which leverage metadata in BIDS format to automatically configure suitable processing workflows (Cieslak et al. 2021).

***Anatomical data preprocessing.*** The T1w image was corrected for intensity non-uniformity (INU) using *N4BiasFieldCorrection* (ANTs v2.3.1) (Tustison et al. 2010) and used as a T1w-reference throughout the workflow. The T1w-reference was then skull-stripped using *antsBrainExtraction.sh* (ANTs v2.3.1), using OASIS as the target template. Spatial normalisation to the ICBM 152 Nonlinear Asymmetrical template version 2009c (Fonov et al. 2009) in the Montreal Neuroimaging Institute space (MNI) was performed through nonlinear registration with *antsRegistration* (ANTs v2.3.1, (Avants et al. 2008), using brain-extracted versions of both T1w volume and template. Brain tissue segmentation of cerebrospinal fluid (CSF), white matter (WM), and grey matter (GM) were performed on the brain-extracted T1w using *FAST* (FSL v6.0.5.1) (Zhang et al. 2001).

***Diffusion data preprocessing*.** MP-PCA denoising, as implemented in MRtrix3’s *dwidenoise* (Veraart et al. 2016) was applied with a 5-voxel window. After MP-PCA, B1 field inhomogeneity was corrected using *dwibiascorrect* from MRtrix3 with the N4 algorithm (Tustison et al. 2010). After B1 bias correction, the mean intensity of the DWI series was adjusted so all the mean intensity of the $b=0$ images matched across each DWI scanning sequence. Motion correction was performed using only $b=0$ images. An unbiased $b=0$ template was constructed over 3 iterations of Affine registrations. The SHORELine method was used to estimate head motion in $b>0$ images (i.e., *3dSHORE*). A total of 2 iterations were run using an Affine transform. Model-generated images were transformed into alignment with each $b>0$ image. Both slicewise and whole-brain QC measures (cross-correlation and R^2^) were calculated. A deformation field to correct susceptibility distortions was estimated based on two EPI references with opposing phase-encoding directions, using *3dQwarp* in AFNI (Cox and Hyde 1997). Based on the estimated susceptibility distortion, an unwarped $b=0$ reference was calculated for a more accurate co-registration with the anatomical reference.

The output from QSIprep labelled by the suffix *_space-T1w_desc-preproc_dwi.nii.gz* was used for further processing. The implementation of the DSIstudio (<https://dsi-studio.labsolver.org/>) workflow (i.e., *dsi_studio_gqi*) in the QSIprep pipeline was utilised to perform diffusion reconstruction, whole-brain tractography, and brain network construction (Yeh et al. 2010; Cieslak et al. 2021). Diffusion orientation distribution functions (ODFs) were reconstructed in both native space and template space (i.e., MNI ICBM152 based on q-space diffeomorphic reconstruction) using generalised q-sampling imaging with a ratio of mean diffusion distance of 1.25 (Yeh et al. 2010). The GQI is a model-free method for quantifying the density of water diffusion in various orientations while providing directional and quantitative information regarding crossing fibres (Yeh et al. 2010, 2013).

***Quality control (QC).*** The anatomical and dMRI data were initially inspected for QC assurance using QSIprep’s visual reports (Cieslak et al. 2021). All data were visually assessed for accurate alignment of T1w images and noticeable signal dropouts. The QC of dMRI data was performed using QSIprep’s QC report, as described in (Yeh et al. 2019). Based on this QC, image dimensions, image resolutions, DWI count, and b-table were checked for consistency within the dataset. Then, the diffusion image quality metric was evaluated by neighbouring DWI correlation (NDC), which summarises the pairwise spatial correlation between each pair of volumes that sample the closest points in q-space (Yeh et al. 2019; Cieslak et al. 2021). The NDC with lower values represents decreased data quality derived from misalignment between volumes, prominent eddy current artefacts, head motion artefacts, or any head coil issues that might impact the diffusion signals (Yeh et al. 2019). Based on NDC scores, the QC procedure applies the outlier checking function (i.e., 3 median absolute deviations) to label noisy data with *Low-quality outlier* labels (Yeh et al. 2019). For our dataset, the NDC values were high and similar across all dMRI volumes (0.906 ± 0.008). Only four outlier scans were identified based on corrected NDC scores (N_APD_=3, N_HC_=1).

## Network construction

Whole-brain fibre tracking for each participant was performed using a deterministic tractography using a modified fibre assessment by continuous tracking (FACT) algorithm (Mori et al. 1999; Mori and van Zijl 2002). Five million streamlines were reconstructed, and tracts less than 10 mm and greater than 400 mm were eliminated. For the fibre tracking, the angular threshold of 45 degrees and step size of 0.94 mm were set. The quantitative anisotropy (QA) scalar was then calculated along the path of each reconstructed streamline. The QA is an index of GQI, a measure of anisotropic spins that diffuse along the fibre orientation (Yeh et al. 2010). In other words, QA is the density of anisotropic diffusing water after removing the isotropic components. Along with QA, fractional anisotropy and generalised FA were also calculated. Compared to FA and GFA, the QA is less sensitive to the partial volume effect, and it can improve deterministic tractography by filtering noisy fibres and defining track terminations (Yeh et al. 2013). For the construction of the brain network, brain nodes were defined according to Brainnetome parcellation (<http://atlas.brainnetome.org/>) with 210 cortical and 36 subcortical regions (246 regions), which provides a fine-grained, cross-validated atlas and contains information on both anatomical and functional connections (Fan et al. 2016). Network edges were defined where at least one track connected a pair of regions. This connectivity was determined where connecting track passed through the parcellated areas (i.e., connectivity type in DSIstudio: *pass*). Then edge weights were computed based on the mean QA along tracks connecting any pair of regions of interest (ROIs). The network construction resulted in an individual-specific symmetric undirected weighted connectivity matrix with dimensions of $246\times246$. In addition to QA-weighted networks, supplemental analyses were carried out to assess the robustness of results on edge weights of every pair of regions defined as mean FA (i.e., *dti-fa*) and mean generalised FA (i.e., *gfa*). Additionally, automated anatomical labelling parcellation (Tzourio-Mazoyer et al. 2002) was used for constructing brain structural network to validate the results.

## Connectome analysis

Graph measures for each participant were computed on the undirected weighted structural network to investigate microstructural changes in the brain structural topology. Graph theory analysis was carried out using the brain connectivity toolbox (BCT, version 03/03/2019, <https://sites.google.com/site/bctnet/>) on MATLAB R2019b (<https://mathworks.com/>).

***Edge-wise connectivity.*** The network-based statistic (NBS) approach (Zalesky et al. 2010) was conducted on an individual’s structural matrices to assess the between-group differences on the edge-wise connection. The NBS is a nonparametric statistical method that controls family-wise error (FWE) to identify the largest connected component in the form of alteration. Initially, a primary statistical threshold ($p<0.05$, uncorrected) was used to identify connected components and their sizes based on a set of supra-threshold links. Second, to test the significance of each identified connected sub-networks, the empirical null distribution of component size was evaluated using a nonparametric permutation test with 10000 randomisations. Afterwards, two-sample t-tests were performed for each pairwise connection linking 246 brain regions to test group differences in structural connectivity in either direction (two-tailed hypothesis test, Initial *t* threshold = 3.9805). Age was controlled as a covariate.

***Rich-club organisation.*** The rich-club architecture in a network exists when hub nodes are highly connected, more so than expected by chance (Zhou and Mondragon 2004; van den Heuvel and Sporns 2011; Fornito et al. 2016a). This tendency of hubs to be highly linked with each other can be defined by calculating the rich-club coefficient. In the present study, the weighted rich-club coefficient, $\varphi^{W}\left( k \right)$ (BCT function: *rich_club_wu*), was computed on the group-averaged QA-weighted structural network based on the following equations (Opsahl et al. 2008):

$$\varphi^{W}\left( k \right)=\frac{W_{>k}}{\sum_{l=1}^{E_{>k}} W_{l}^{ranked}}$$

where $k$ is the degree of a node, $E_{>k}$ denotes the number of edges that exists in the subgraph with degree $>k$, $W_{>k}$ is the sum of the weights on edges within the subgraph of nodes with a rank greater than $k$, and $W^{ranked}$ represents a vector of edge weights from highest to lowest across the entire network. $\varphi^{W}\left( k \right)$ indicates the rich-club effect for each level $k$ in the empirical (i.e., observed) network.

Random networks (e.g., Erdős-Rényi model) show characteristics of $\varphi^{W}\left( k \right)$ by increasing $k$ in the network, where nodes with a higher degree are more likely to be interconnected with each other by chance, hence $\varphi^{W}\left( k \right)$ is normalised with a set from a comparable random network to address this problem (Colizza et al. 2006; McAuley et al. 2007; van den Heuvel and Sporns 2011; Fornito et al. 2016a). In the current study, 1000 randomised networks were constructed by shuffling the weighted links in the group-averaged network while preserving the degree distribution and sequence of the matrix (BCT function: *randmio_und*, 50 iterations) (Maslov and Sneppen 2002). The rich-club coefficient was then computed for each random network, $\varphi_{rand}^{W}(k)$ and, for each level $k$, the normalised rich-club coefficient, $\varphi_{norm}^{W}(k)$, was computed as the ratio between $\varphi^{W}\left( k \right)$ and average of $\varphi_{rand}^{W}(k)$

across 1000 networks (Colizza et al. 2006):

$$\varphi_{norm}(k)=\frac{\varphi(k)}{\varphi_{rand}(k)}$$

where for each level $k$, $\varphi_{norm}^{W}(k)>1$ suggests the existence of a rich-club organisation in the network (Colizza et al. 2006; Fornito et al. 2016a). A permutation test was used to evaluate the statistical significance of rich-club organisation based on the empirical null distribution of 1000 randomised networks (Bassett and Bullmore 2009; van den Heuvel and Sporns 2011). Then, for the range of $k$ expressing rich-club organisation, one-tailed permutation tests determine whether $\varphi^{W}\left( k \right)$ is significantly greater than $\varphi_{rand}^{W}(k)$ at each level of $k$ (one-tailed, $p<0.05$, 10000 shuffling). This analysis verifies whether any apparent rich-club organisation compared to the random topology significantly exceeds 1, indicating the existence of the rich-club organisation (van den Heuvel and Sporns 2011; Barker et al. 2017)

Brain hub regions were identified according to the consensus-based definition of hubs in structural connectivity networks (van den Heuvel et al. 2010), whereby hubs are defined as nodes with high nodal strength (NS), high betweenness centrality (BC), low average path length (APL) and low clustering coefficient (CC). More information regarding the definition of the graph measures utilised in this study can be found in (Rubinov and Sporns 2010). NS, BC, APL and CC were computed to identify brain hubs based on the group-averaged matrices. Each region was then assigned a score based on the following conditions: (1) the top 20% of regions with the highest NS ($k>$ 1 standard deviation above the mean); (2) the top 20% of regions with the highest BC; (3) the bottom 20% of regions with lowest APL; or (4) bottom 20% of regions with lowest CC. Based on these scores (i.e., 0 - 4), ROIs with the highest score (i.e., $\geq3$) that consistently exist in both groups were selected as brain hubs (van den Heuvel et al. 2010; Fornito et al. 2016b).

Following hub detection, brain regions were classified into core regions (i.e., hub, also known as rich-club regions) and peripheral regions (i.e., non-hub regions). Categorization of brain regions allowed for defining the connections between brain regions into three classes: *rich* (links between core regions), *feeder* (links between the core and peripheral regions) and *local* (links between peripheral areas). For each connectome, the connectivity strength of *rich*, *feeder* and *local* connections was then computed as the sum of all edge weights within each connection class:

$$\sum_{Rich/Feeder/Local} edge weights$$

**Supplementary results**

**
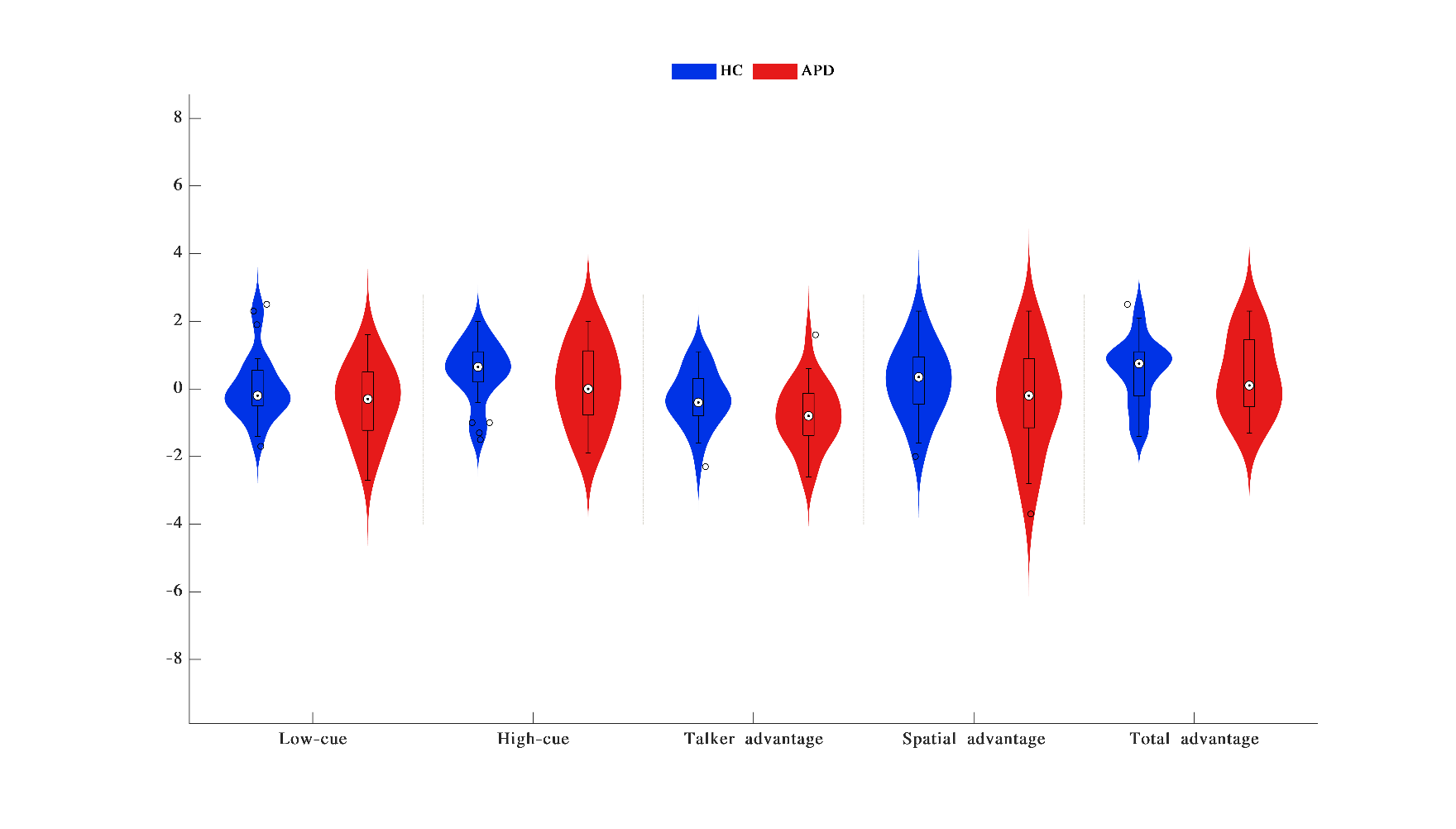
Figure S1** Distribution of LiSN-S z-scores (Low-cue, High-cue, Talker advantage, Spatial advantage, Total advantage) for APD and HC participants. HC – healthy control, APD – auditory processing disorder, LiSN-S - listening-in-spatialised-noise-sentences.

**Table S1** Results from between-group differences based on FA and GFA connectivity measures

| **Connectivity** | | **Metric** | **ROI** | | ***p-*value** | ***t* stat** | **FDR** | **Bonferroni** |
| --- | --- | --- | --- | --- | --- | --- | --- | --- |
| FA | APL | | | 144 | 0.0003 | 3.9764 | 0.165 | 0.0653 |
|  | BC | | | 64 | 0.0001 | 3.9805 | 0.11 | 0.0644 |
| GFA | APL | | | 144 | 0.0003 | 3.9048 | 0.165 | 0.0827 |
|  | BC | | | 64 | 0.0001 | 3.8764 | 0.165 | 0.0907 |

***Note:*** ROI – region of interest, *t* stat – test statistic, FA – fractional anisotropy, GFA – generalized fractional anisotropy, APL – average path length, BC – betweenness centrality, FDR – false discovery rate.

**Table S2** Results from between-group differences in the APL and BC metrics based on AAL parcellation

|  | **APL** | | | **BC** | | |
| --- | --- | --- | --- | --- | --- | --- |
| **ROIs** | ***p*-value** | **t value** | **Bonferroni** | ***p*-value** | ***t*-value** | **Bonferroni** |
| 1 | 0.0608 | 1.5707 | 0.9999 | 0.3816 | 0.2812 | 1 |
| 2 | 0.0114 | 2.3166 | 0.8648 | 0.843 | -1.0338 | 1 |
| 3 | 0.05 | 1.6671 | 0.9998 | 0.5274 | -0.0588 | 1 |
| 4 | 0.0396 | 1.782 | 0.9989 | 0.4915 | 0.0143 | 1 |
| 5 | 0.0166 | 2.1721 | 0.9386 | 0.1999 | 0.857 | 1 |
| 6 | 0.007 | 2.4627 | 0.7578 | 0.5416 | -0.1184 | 1 |
| 7 | 0.0152 | 2.2269 | 0.9159 | 0.1794 | 0.9548 | 1 |
| 8 | 0.0648 | 1.5407 | 0.9999 | 0.1525 | 1.0814 | 1 |
| 9 | 0.0054 | 2.5321 | 0.7003 | 0.0462 | 1.7285 | 0.9994 |
| 10 | 0.0067 | 2.5077 | 0.7218 | 0.8925 | -1.277 | 1 |
| 11 | 0.0495 | 1.6834 | 0.9997 | 0.6928 | -0.4943 | 1 |
| 12 | 0.0503 | 1.65 | 0.9998 | 0.4654 | 0.0903 | 1 |
| 13 | 0.0265 | 1.9728 | 0.9866 | 0.7449 | -0.6493 | 1 |
| 14 | 0.0372 | 1.7897 | 0.9986 | 0.327 | 0.466 | 1 |
| 15 | 0.1143 | 1.213 | 1 | 0.2066 | 0.8562 | 1 |
| 16 | 0.0089 | 2.4222 | 0.7921 | 0.8123 | -0.9867 | 1 |
| 17 | 0.0724 | 1.4925 | 1 | 0.4967 | -0.0005 | 1 |
| 18 | 0.1141 | 1.2063 | 1 | 0.0241 | 1.8778 | 0.9952 |
| 19 | 0.0507 | 1.6474 | 0.9998 | 0.1045 | 1.2946 | 1 |
| 20 | 0.0226 | 2.0129 | 0.9811 | 0.5338 | -0.0836 | 1 |
| 21 | 0.1626 | 0.9881 | 1 | 0.8426 | -1.0456 | 1 |
| 22 | 0.0199 | 2.09 | 0.966 | 0.8034 | -0.8594 | 1 |
| 23 | 0.0486 | 1.6682 | 0.9998 | 0.131 | 1.1498 | 1 |
| 24 | 0.0522 | 1.6455 | 0.9998 | 0.255 | 0.6572 | 1 |
| 25 | 0.0146 | 2.2193 | 0.9194 | 0.4423 | 0.1752 | 1 |
| 26 | 0.0044 | 2.6645 | 0.5857 | 0.5751 | -0.218 | 1 |
| 27 | 0.0034 | 2.8119 | 0.4569 | 0.8163 | -0.9552 | 1 |
| 28 | 0.0044 | 2.7063 | 0.5479 | 0.4265 | 0.1959 | 1 |
| 29 | 0.0934 | 1.3296 | 1 | 0.7717 | -0.7466 | 1 |
| 30 | 0.0149 | 2.2037 | 0.9264 | 0.05 | 1.6668 | 0.9998 |
| 31 | 0.0665 | 1.5081 | 1 | 0.548 | -0.1628 | 1 |
| 32 | 0.0172 | 2.113 | 0.9597 | 0.5313 | -0.0894 | 1 |
| 33 | 0.043 | 1.727 | 0.9994 | 0.4737 | 0.0832 | 1 |
| 34 | 0.0368 | 1.7945 | 0.9986 | 0.0283 | 1.9353 | 0.9909 |
| 35 | 0.0019 | 2.9761 | 0.3322 | 0.9469 | -1.6252 | 1 |
| 36 | 0.0437 | 1.7167 | 0.9996 | 0.7372 | -0.659 | 1 |
| 37 | 0.0016 | 3.0439 | 0.2858 | 0.6674 | -0.4417 | 1 |
| 38 | 0.0051 | 2.6044 | 0.6375 | 0.8411 | -1.0149 | 1 |
| 39 | 0.0132 | 2.2322 | 0.9129 | 0.0323 | 1.9039 | 0.9934 |
| 40 | 0.0042 | 2.6831 | 0.5698 | 0.5576 | -0.1792 | 1 |
| 41 | 0.0148 | 2.2247 | 0.9169 | 0.1014 | 1.2988 | 1 |
| 42 | 0.0194 | 2.1135 | 0.9595 | 0.2325 | 0.7325 | 1 |
| 43 | 0.025 | 1.9805 | 0.9855 | 0.296 | 0.5449 | 1 |
| 44 | 0.0435 | 1.7316 | 0.9994 | 0.0061 | 2.5594 | 0.6772 |
| 45 | 0.0034 | 2.742 | 0.5158 | 0.6881 | -0.4847 | 1 |
| 46 | 0.0215 | 2.0399 | 0.9768 | 0.3558 | 0.3823 | 1 |
| 47 | 0.0056 | 2.5885 | 0.6522 | 0.7613 | -0.7065 | 1 |
| 48 | 0.004 | 2.7475 | 0.5115 | 0.7794 | -0.7883 | 1 |
| 49 | 0.0217 | 2.0279 | 0.9784 | 0.3034 | 0.523 | 1 |
| 50 | 0.093 | 1.3465 | 1 | 0.2505 | 0.6765 | 1 |
| 51 | 0.0896 | 1.3559 | 1 | 0.2585 | 0.6407 | 1 |
| 52 | 0.0356 | 1.8421 | 0.997 | 0.329 | 0.4445 | 1 |
| 53 | 0.1293 | 1.1533 | 1 | 0.5843 | -0.3387 | 1 |
| 54 | 0.0505 | 1.6778 | 0.9998 | 0.4608 | 0.1314 | 1 |
| 55 | 0.0098 | 2.4008 | 0.8081 | 0.7922 | -0.8273 | 1 |
| 56 | 0.0013 | 3.1072 | 0.2447 | 0.9473 | -1.64 | 1 |
| 57 | 0.0239 | 2.004 | 0.9824 | 0.9034 | -1.3238 | 1 |
| 58 | 0.0143 | 2.216 | 0.9206 | 0.8908 | -1.245 | 1 |
| 59 | 0.0014 | 3.004 | 0.312 | 0.955 | -1.7241 | 1 |
| 60 | 0.0576 | 1.6137 | 0.9999 | 0.6306 | -0.3529 | 1 |
| 61 | 0.1548 | 1.0116 | 1 | 0.6674 | -0.4668 | 1 |
| 62 | 0.035 | 1.8437 | 0.997 | 0.6905 | -0.6177 | 1 |
| 63 | 0.0334 | 1.8585 | 0.9963 | 0.7457 | -0.7008 | 1 |
| 64 | 0.0037 | 2.6927 | 0.5605 | 0.4859 | 0.0411 | 1 |
| 65 | 0.2664 | 0.6327 | 1 | 0.2895 | 0.5687 | 1 |
| 66 | 0.0075 | 2.5142 | 0.7162 | 0.259 | 0.6991 | 1 |
| 67 | 0.0087 | 2.4249 | 0.7901 | 0.649 | -0.3789 | 1 |
| 68 | 0.0323 | 1.8574 | 0.9963 | 0.2974 | 0.5398 | 1 |
| 69 | 0.0065 | 2.5047 | 0.7238 | 0.8713 | -1.146 | 1 |
| 70 | 0.0181 | 2.1208 | 0.9572 | 0.1883 | 1.0314 | 1 |
| 71 | 0.0123 | 2.2826 | 0.8847 | 0.3151 | 0.4946 | 1 |
| 72 | 0.0181 | 2.1236 | 0.9566 | 0.4082 | 0.2408 | 1 |
| 73 | 0.0739 | 1.4546 | 1 | 0.4726 | 0.0528 | 1 |
| 74 | 0.0362 | 1.8389 | 0.9971 | 0.7287 | -0.6178 | 1 |
| 75 | 0.1542 | 1.0276 | 1 | 0.1333 | 1.1346 | 1 |
| 76 | 0.0068 | 2.5044 | 0.724 | 0.8594 | -1.0794 | 1 |
| 77 | 0.0181 | 2.1054 | 0.9628 | 0.7047 | -0.5423 | 1 |
| 78 | 0.0181 | 2.1209 | 0.9571 | 0.6471 | -0.363 | 1 |
| 79 | 0.274 | 0.5953 | 1 | 0.3667 | 0.5735 | 1 |
| 80 | 0.0066 | 2.5339 | 0.699 | 0 | 0 | 0 |
| 81 | 0.142 | 1.078 | 1 | 0.6714 | -0.5248 | 1 |
| 82 | 0.0024 | 2.8988 | 0.3885 | 0.3131 | 0.4776 | 1 |
| 83 | 0.0359 | 1.8235 | 0.9976 | 0.5315 | -0.0856 | 1 |
| 84 | 0.0098 | 2.3567 | 0.8391 | 0.2434 | 0.7088 | 1 |
| 85 | 0.1447 | 1.0659 | 1 | 0.5626 | -0.1718 | 1 |
| 86 | 0.0549 | 1.6161 | 0.9999 | 0.2114 | 0.8088 | 1 |
| 87 | 0.009 | 2.4559 | 0.7648 | 0.326 | 0.4566 | 1 |
| 88 | 0.0275 | 1.9696 | 0.987 | 0.427 | 0.1731 | 1 |
| 89 | 0.0187 | 2.1146 | 0.9591 | 0.9041 | -1.3137 | 1 |
| 90 | 0.0009 | 3.2492 | 0.1759 | 0.52 | -0.0604 | 1 |
| 91 | 0.0488 | 1.6664 | 0.9998 | 0.0946 | 1.3502 | 1 |
| 92 | 0.1951 | 0.876 | 1 | 0.1148 | 1.2099 | 1 |
| 93 | 0.0768 | 1.4422 | 1 | 0.0759 | 1.4581 | 1 |
| 94 | 0.1857 | 0.8953 | 1 | 0.1444 | 1.1127 | 1 |
| 95 | 0.1067 | 1.2505 | 1 | 0.2251 | 0.7687 | 1 |
| 96 | 0.249 | 0.673 | 1 | 0.3375 | 0.4123 | 1 |
| 97 | 0.0242 | 1.9954 | 0.9836 | 0.5468 | -0.1299 | 1 |
| 98 | 0.0336 | 1.8318 | 0.9973 | 0.4268 | 0.1861 | 1 |
| 99 | 0.0045 | 2.6885 | 0.5644 | 0.8689 | -1.1299 | 1 |
| 100 | 0.0673 | 1.5162 | 1 | 0.4317 | 0.1743 | 1 |
| 101 | 0.1422 | 1.0719 | 1 | 0.699 | -0.5183 | 1 |
| 102 | 0.1551 | 1.0197 | 1 | 0.6695 | -0.5431 | 1 |
| 103 | 0.0767 | 1.4356 | 1 | 0.549 | -0.103 | 1 |
| 104 | 0.0945 | 1.336 | 1 | 0.7371 | -0.659 | 1 |
| 105 | 0.1489 | 1.0745 | 1 | 0.8303 | -0.974 | 1 |
| 106 | 0.064 | 1.5529 | 0.9999 | 0.965 | -1.8449 | 1 |
| 107 | 0 | 0 | 0 | 0.3609 | 0.5724 | 1 |
| 108 | 0.1081 | 1.258 | 1 | 0.1898 | 0.9952 | 1 |
| 109 | 0.2109 | 0.7997 | 1 | 0.8272 | -0.9451 | 1 |
| 110 | 0.0997 | 1.3069 | 1 | 0.374 | 0.3243 | 1 |
| 111 | 0.0291 | 1.9469 | 0.9898 | 0.1331 | 1.1351 | 1 |
| 112 | 0.0106 | 2.3272 | 0.8579 | 0.9254 | -1.4333 | 1 |
| 113 | 0.0464 | 1.6894 | 0.9997 | 0.978 | -2.0358 | 1 |
| 114 | 0.1612 | 0.9923 | 1 | 0.0437 | 1.7413 | 0.9994 |
| 115 | 0.1812 | 0.9489 | 1 | 0.5047 | -0.0162 | 1 |
| 116 | 0.0829 | 1.409 | 1 | 0.3532 | 0.4187 | 1 |

***Note:*** AAL – automated anatomical labelling, APL- average path length, BC – betweenness centrality.

**
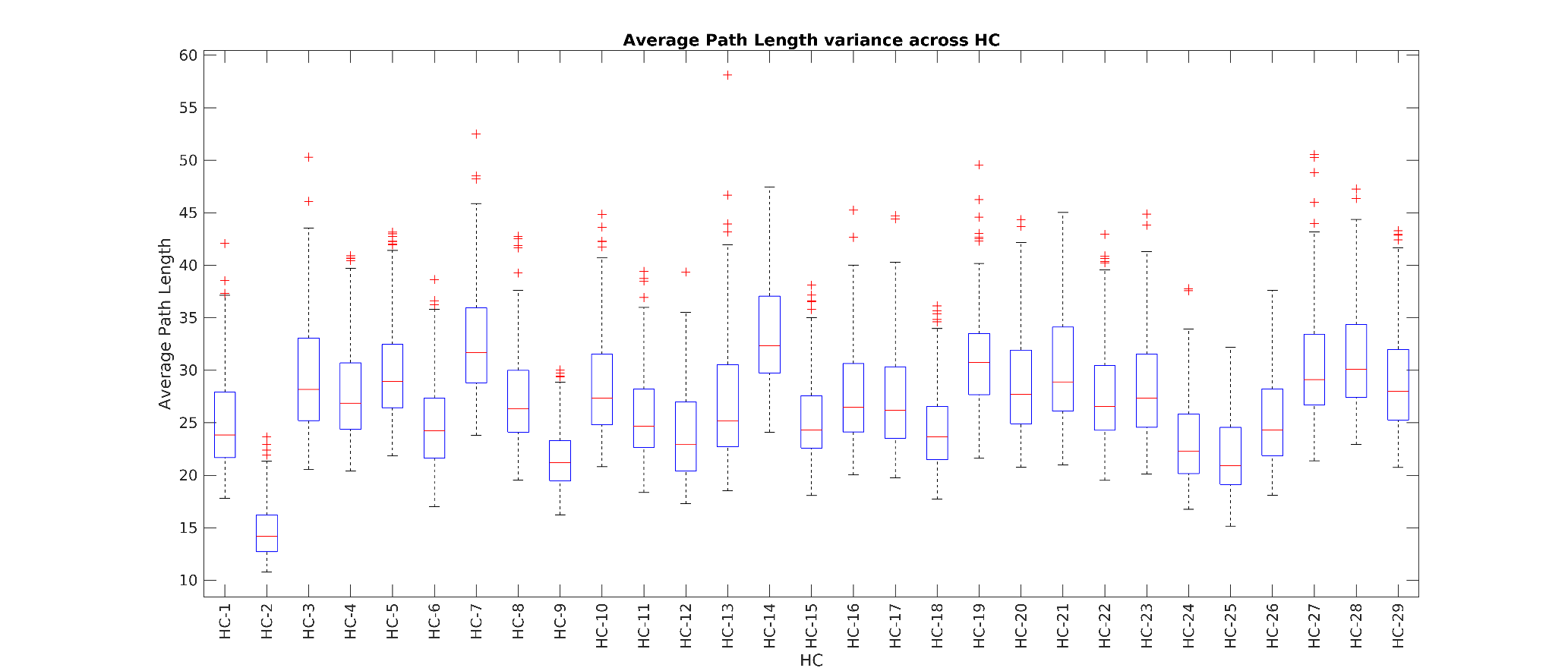

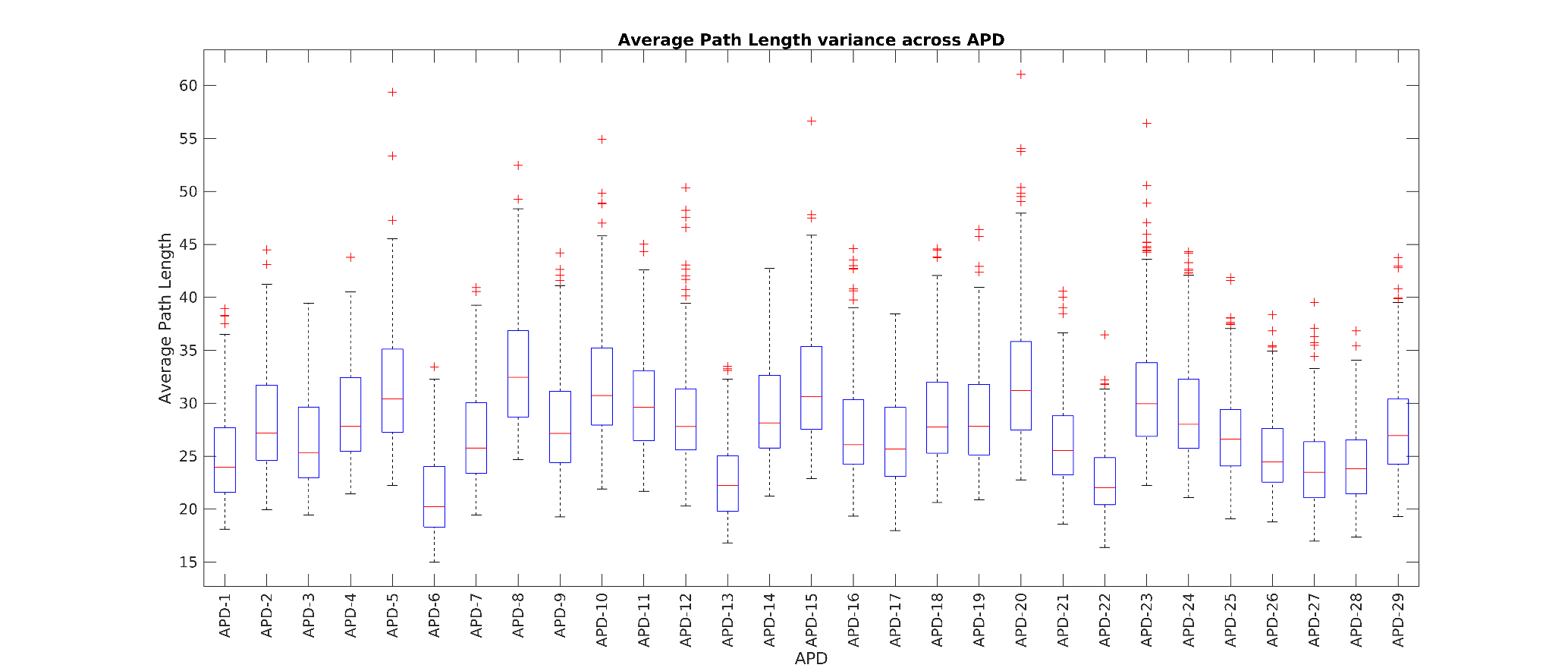
Figure S2** Data distribution of average path length across APD individuals. APD – auditory processing disorder.

**Figure S3** Data distribution of average path length across HC individuals. HC – healthy control.

**
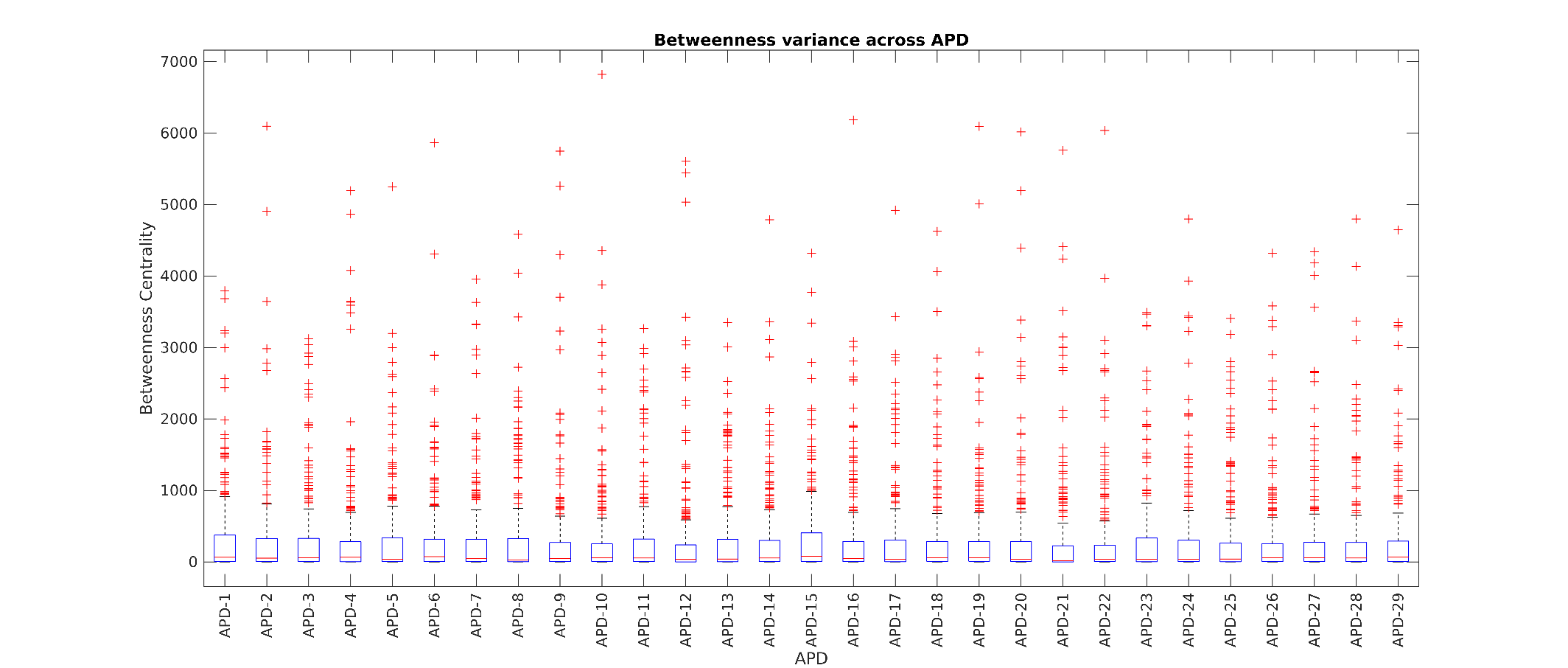

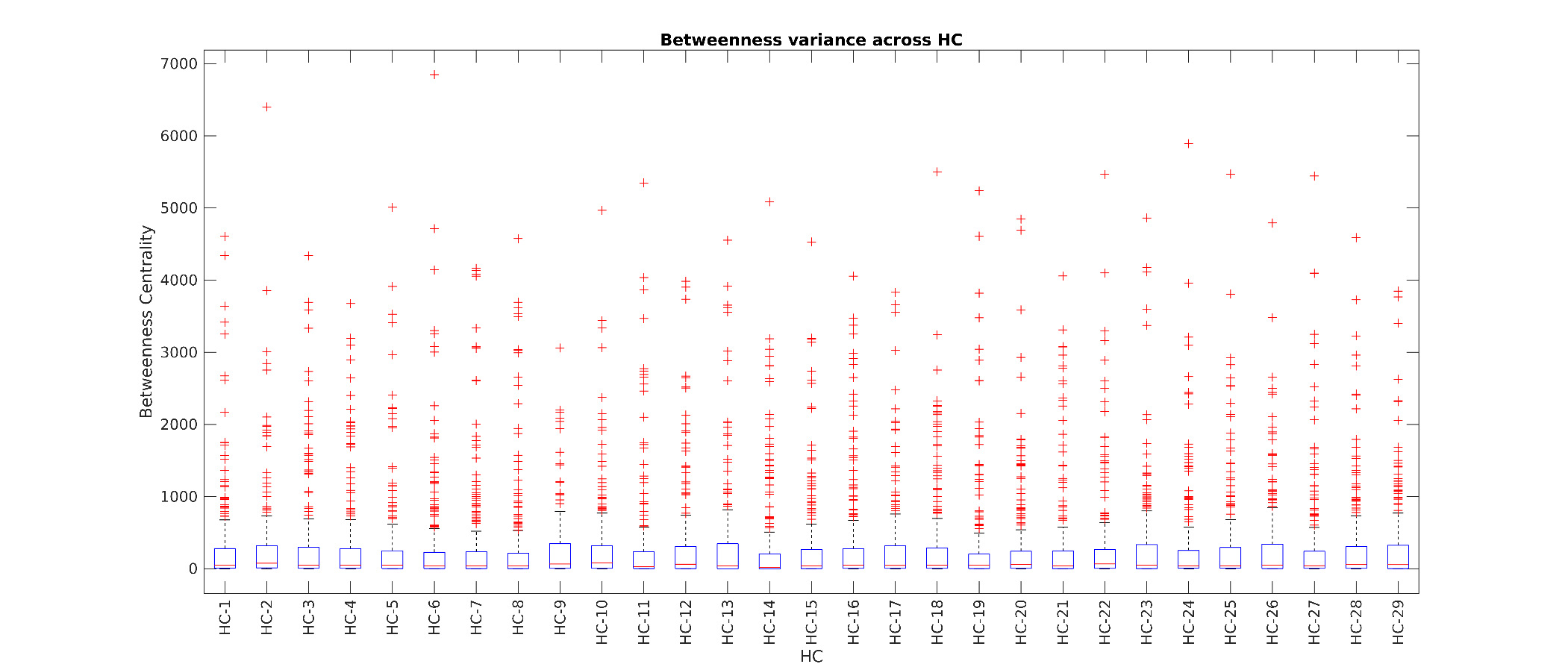
Figure S4** Data distribution of betweenness centrality across APD individuals. APD – auditory processing disorder.

**Figure S5** Data distribution of betweenness centrality across HC individuals. HC – healthy control.

**References**

Abraham A, Pedregosa F, Eickenberg M, Gervais P, Mueller A, Kossaifi J, Gramfort A, Thirion B, Varoquaux G. 2014. Machine learning for neuroimaging with scikit-learn. Front Neuroinform. 8:14.

Avants BB, Epstein CL, Grossman M, Gee JC. 2008. Symmetric diffeomorphic image registration with cross-correlation: evaluating automated labeling of elderly and neurodegenerative brain. Med Image Anal. 12:26–41.

Barker MD, Kuruvilla-Mathew A, Purdy SC. 2017. Cortical Auditory-Evoked Potential and Behavioral Evidence for Differences in Auditory Processing between Good and Poor Readers. J Am Acad Audiol. 28:534–545.

Bassett D, Bullmore ET. 2009. Human brain networks in health and disease. Curr Opin Neurol. 22:340–347.

Cieslak M, Cook PA, He X, Yeh F-C, Dhollander T, Adebimpe A, Aguirre GK, Bassett DS, Betzel RF, Bourque J, Cabral LM, Davatzikos C, Detre JA, Earl E, Elliott MA, Fadnavis S, Fair DA, Foran W, Fotiadis P, Garyfallidis E, Giesbrecht B, Gur RC, Gur RE, Kelz MB, Keshavan A, Larsen BS, Luna B, Mackey AP, Milham MP, Oathes DJ, Perrone A, Pines AR, Roalf DR, Richie-Halford A, Rokem A, Sydnor VJ, Tapera TM, Tooley UA, Vettel JM, Yeatman JD, Grafton ST, Satterthwaite TD. 2021. QSIPrep: an integrative platform for preprocessing and reconstructing diffusion MRI data. Nat Methods. 1–4.

Colizza V, Flammini A, Serrano MA, Vespignani A. 2006. Detecting rich-club ordering in complex networks. Nat Phys. 2:110–115.

Cox RW, Hyde JS. 1997. Software tools for analysis and visualization of fMRI data. NMR Biomed. 10:171–178.

Esteban O, Markiewicz CJ, Burns C, Goncalves M, Jarecka D, Ziegler E, Berleant S, Ellis DG, Pinsard B, Madison C, Waskom M, Notter MP, Clark D, Manhães-Savio A, Clark D, Jordan K, Dayan M, Halchenko YO, Loney F, Salo T, Dewey BE, Johnson H, Bougacha S, Keshavan A, Yvernault B, Hamalainen C, Christian H, Ćirić R, Dubois M, Joseph M, Cipollini B, Tilley S II, Visconti di Oleggio Castello M, De La Vega A, Wong J, Kaczmarzyk J, Huntenburg JM, Clark MG, Benderoff E, Erickson D, Dias M de F, Kent JD, Hanke M, Giavasis S, Moloney B, Nichols BN, Tungaraza R, Dell’Orco A, Frohlich C, Wassermann D, de Hollander G, Koudoro S, Eshaghi A, Millman J, Mancini M, Close T, Nielson DM, Varoquaux G, Waller L, Watanabe A, Mordom D, Guillon J, Robert-Fitzgerald T, Chetverikov A, Rokem A, Acland B, Forbes J, Markello R, Gillman A, Bernardoni F, Kong X-Z, Geisler D, Salvatore J, Gramfort A, Doll A, Buchanan C, DuPre E, Liu S, Schaefer A, Kleesiek J, Sikka S, Schwartz Y, Ghayoor A, Lee JA, Mattfeld A, Richie-Halford A, Liem F, Perez-Guevara MF, Heinsfeld AS, Haselgrove C, Durnez J, Lampe L, Poldrack R, Glatard T, Baratz Z, Tabas A, Cumba C, Pérez-García F, Blair R, Iqbal S, Welch D, Contier O, Triplett W, Craddock RC, Correa C, Papadopoulos Orfanos D, Stadler J, Warner J, Sisk LM, Falkiewicz M, Sharp P, Rothmei S, Kim S, Weinstein A, Kahn AE, Kastman E, Bottenhorn K, Grignard M, Perkins LN, Zhou D, Bielievtsov D, Ben-Zvi G, Cooper G, Stojic H, Hui Qian T, Linkersdörfer J, Renfro M, Hinds O, Stanley O, Küttner R, Pauli WM, Xie X, Glen D, Kimbler A, Meyers B, Tarbert C, Ginsburg D, Haehn D, Margulies DS, Condamine E, Ma F, Malone IB, Snoek L, Brett M, Cieslak M, Hallquist M, Molina-Romero M, Bilgel M, Lee N, Kuntke P, Jalan R, Inati S, Gerhard S, Mathotaarachchi S, Saase V, Van A, Steele CJ, Ort E, Lerma-Usabiaga G, Schwabacher I, Arias J, Lai J, Pellman J, Huguet J, Junhao WEN, Leinweber K, Chawla K, Weninger L, Modat M, Mukhometzianov R, Harms R, Andberg SK, Matsubara K, González Orozco AA, Routier A, Marina A, Davison A, Floren A, Park A, Cheung B, McDermottroe C, McNamee D, Shachnev D, Vogel D, Flandin G, Jones H, Gonzalez I, Varada J, Schlamp K, Podranski K, Huang L, Noel M, Pannetier N, Numssen O, Khanuja R, Urchs S, Nickson T, Huang L, Broderick W, Tambini A, Mihai PG, Gorgolewski KJ, Ghosh S. 2022. Nipy/nipype: 1.7.1.

Fan L, Li H, Zhuo J, Zhang Y, Wang J, Chen L, Yang Z, Chu C, Xie S, Laird AR, Fox PT, Eickhoff SB, Yu C, Jiang T. 2016. The Human Brainnetome Atlas: A New Brain Atlas Based on Connectional Architecture. Cereb Cortex. 26:3508–3526.

Fonov VS, Evans AC, McKinstry RC, Almli CR, Collins DL. 2009. Unbiased nonlinear average age-appropriate brain templates from birth to adulthood. Neuroimage. 47:S102.

Fornito A, Zalesky A, Bullmore ET (Eds.). 2016a. Chapter 6 - Components, Cores, and Clubs. In: Fundamentals of Brain Network Analysis. San Diego: Academic Press. p. 163–206.

Fornito A, Zalesky A, Bullmore ET (Eds.). 2016b. Chapter 5 - Centrality and Hubs. In: Fundamentals of Brain Network Analysis. San Diego: Academic Press. p. 137–161.

Garyfallidis E, Brett M, Amirbekian B, Rokem A, van der Walt S, Descoteaux M, Nimmo-Smith I, Dipy Contributors. 2014. Dipy, a library for the analysis of diffusion MRI data. Front Neuroinform. 8:8.

Gorgolewski, Burns CD, Madison C, Clark D, Halchenko YO, Waskom ML, Ghosh SS. 2011. Nipype: a flexible, lightweight and extensible neuroimaging data processing framework in python. Front Neuroinform. 5:13.

Gorgolewski KJ, Alfaro-Almagro F, Auer T, Bellec P, Capotă M, Chakravarty MM, Churchill NW, Cohen AL, Craddock RC, Devenyi GA, Eklund A, Esteban O, Flandin G, Ghosh SS, Guntupalli JS, Jenkinson M, Keshavan A, Kiar G, Liem F, Raamana PR, Raffelt D, Steele CJ, Quirion P-O, Smith RE, Strother SC, Varoquaux G, Wang Y, Yarkoni T, Poldrack RA. 2017. BIDS apps: Improving ease of use, accessibility, and reproducibility of neuroimaging data analysis methods. PLoS Comput Biol. 13:e1005209.

Maslov S, Sneppen K. 2002. Specificity and stability in topology of protein networks. Science. 296:910–913.

McAuley JJ, da Fontoura Costa L, Caetano TS. 2007. Rich-club phenomenon across complex network hierarchies. Appl Phys Lett. 91:084103.

Mori S, Crain BJ, Chacko VP, van Zijl PC. 1999. Three-dimensional tracking of axonal projections in the brain by magnetic resonance imaging. Ann Neurol. 45:265–269.

Mori S, van Zijl PCM. 2002. Fiber tracking: principles and strategies - a technical review. NMR Biomed. 15:468–480.

Opsahl T, Colizza V, Panzarasa P, Ramasco JJ. 2008. Prominence and control: the weighted rich-club effect. Phys Rev Lett. 101:168702.

Rorden C, Karnath H-O, Bonilha L. 2007. Improving Lesion-Symptom Mapping. J Cogn Neurosci. 19:1081–1088.

Rubinov M, Sporns O. 2010. Complex network measures of brain connectivity: uses and interpretations. Neuroimage. 52:1059–1069.

Tustison NJ, Avants BB, Cook PA, Zheng Y, Egan A, Yushkevich PA, Gee JC. 2010. N4ITK: improved N3 bias correction. IEEE Trans Med Imaging. 29:1310–1320.

Tzourio-Mazoyer N, Landeau B, Papathanassiou D, Crivello F, Etard O, Delcroix N, Mazoyer B, Joliot M. 2002. Automated anatomical labeling of activations in SPM using a macroscopic anatomical parcellation of the MNI MRI single-subject brain. Neuroimage. 15:273–289.

van den Heuvel MP, Mandl RCW, Stam CJ, Kahn RS, Hulshoff Pol HE. 2010. Aberrant frontal and temporal complex network structure in schizophrenia: a graph theoretical analysis. J Neurosci. 30:15915–15926.

van den Heuvel MP, Sporns O. 2011. Rich-club organization of the human connectome. J Neurosci. 31:15775–15786.

Veraart J, Novikov DS, Christiaens D, Ades-Aron B, Sijbers J, Fieremans E. 2016. Denoising of diffusion MRI using random matrix theory. Neuroimage. 142:394–406.

Yeh F-C, Verstynen TD, Wang Y, Fernández-Miranda JC, Tseng W-YI. 2013. Deterministic diffusion fiber tracking improved by quantitative anisotropy. PLoS One. 8:e80713.

Yeh F-C, Wedeen VJ, Tseng W-YI. 2010. Generalized q-sampling imaging. IEEE Trans Med Imaging. 29:1626–1635.

Yeh F-C, Zaydan IM, Suski VR, Lacomis D, Richardson RM, Maroon JC, Barrios-Martinez J. 2019. Differential tractography as a track-based biomarker for neuronal injury. Neuroimage. 202:116131.

Zalesky A, Fornito A, Bullmore ET. 2010. Network-based statistic: identifying differences in brain networks. Neuroimage. 53:1197–1207.

Zhang Y, Brady M, Smith S. 2001. Segmentation of brain MR images through a hidden Markov random field model and the expectation-maximization algorithm. IEEE Trans Med Imaging. 20:45–57.

Zhou S, Mondragon RJ. 2004. The rich-club phenomenon in the Internet topology. IEEE Commun Lett. 8:180–182.
